# Systematic Review and Critical Analysis on Dietary Supplements for Male Infertility: From a Blend of Ingredients to a Rationale Strategy

**DOI:** 10.3389/fendo.2021.824078

**Published:** 2022-02-04

**Authors:** Andrea Garolla, Gabriel Cosmin Petre, Francesco Francini-Pesenti, Luca De Toni, Amerigo Vitagliano, Andrea Di Nisio, Giuseppe Grande, Carlo Foresta

**Affiliations:** ^1^ Unit of Andrology and Reproductive Medicine & Centre for Male Gamete Cryopreservation, Department of Medicine, University of Padova, Padova, Italy; ^2^ Department of Medicine, Clinical Nutrition Unit, University of Padova, Padova, Italy; ^3^ Department of Women and Children’s Health, University of Padua, Padua, Italy; ^4^ Unit of Obstetrics and Gynecology, Madonna della Navicella Hospital, Venice, Italy

**Keywords:** fertility, male reproduction, nutraceuticals, semen parameters, supplements

## Abstract

**Content:**

Dietary supplements (DS) for male infertility marketed in Italy were evaluated for composition, concentration of ingredients, and recommended daily dose. A systematic review of literature identified ingredients potentially effective on sperm parameters and their minimal effective daily dose (mED).

**Objective:**

This study was conducted in order to critically evaluate the composition and efficacy of DS marketed in Italy.

**Design, Setting, and Participants:**

This was a systematic review of randomized controlled trials.

**Evidence Acquisition:**

A formula allowed us to classify the expected efficacy of each DS, based on composition. Each DS was scored and included into three classes of expected efficacy: high, low, and none.

**Evidence Synthesis:**

Among 24 supplements, 3 (12.5%) fall in high, 9 (37.5%) in lower, and 12 (50.0%) in no expected efficacy class. DS composition showed 36 substances, 18 with no literature on male fertility and 18 showing positive effect on sperm parameters, thus considered potentially active ingredients (PAI). All DS were mixtures of ingredients, containing from 2 to 17 different substances. Fifteen supplements (65.2%) contained at least 1 ingredient without evidence of efficacy and 21 formulations had PAI dosed below mED. Some PAI were associated to the improvement of specific sperm parameters.

**Conclusions:**

DS were usually blends of many substances that are frequently employed at negligible dose or without any evidence of efficacy on male reproduction. Some ingredients have been demonstrated to be effective on specific sperm parameters by RCTs. We report a list of ingredients with potential efficacy on specific sperm parameters, aimed to allow a tailored use of DS.

**Patient Summary:**

The market of DS for male infertility offers products with potential efficacy in the improvement of sperm parameters but also many with uncertain effects. Based on current scientific literature, our study can help in the choice of DS that are more likely to be effective on specific sperm alterations, so providing the best supplementation for each patient.

## Introduction

Male factor infertility accounts the most varied and multifactorial causes (1, 2). Besides idiopathic infertility (up to 25% of cases), organic causes range from genital tract infections/inflammation, hormonal alterations, varicocele, and genetic problems (3–5). Many recent studies emphasized the role of other risk factors such as incorrect lifestyles, malnutrition, and abuse substances (6, 7). The hypothesis is that these conditions, inducing an elevation of radical oxygen and nitrogen species, might impair spermatogenesis, both directly, through an alteration of a redox status, and indirectly, interfering with the hypothalamic–pituitary–testicular axis (8–10). A recent survey performed by American urologists on clinical practice in the treatment of idiopathic male infertility showed that 64.9% of caregivers use dietary supplements (DS), empirically in the face of a lack of recommendations on the guidelines for the use of these products (11). The European Food Safety Authority (EFSA) stated that “supplements aren’t intended to treat or prevent diseases in humans or to modify physiological functions, but only to support specific physiological functions” (12). Anyway, all meta-analyses and guidelines citing the use of DS for male infertility advise to carefully evaluate the causes of infertility, as well as to accurately assess nutritional status (11, 13).

The term “nutraceutical” is not defined by law and products that fall into this category are mainly contained in the DS (14, 15). In recent years, a growing use of nutraceutical products has been recorded among men seeking fertility or complaining other andrological problems (16, 17). DS are widely available on the market, even if a proven efficacy has not yet been demonstrated for most of them. Despite many authors showing the positive effects of some substances on semen parameters and fertility outcomes (18–20), many others reported the lack of efficacy and even potential side effects (16, 20). We recently summarized the state-of-the-art of single ingredients currently present in the DS marketed to improve sperm parameters (17). The main conclusion was that some DS were mixtures of ingredients with uncertain or unreported benefits and contained substances with very low dosage.

Despite these attempts to clarify the specific role of each ingredient, several confounding factors make difficult and still empirical the choice of the proper formulation for each patient at the right time, in a perspective of personalized medicine. This is due to several issues: i) prescribers rarely know the nutritional state of the patient before administering a nutraceutical product; ii) it is still unclear which infertile patient may have beneficial effects from nutraceutical substances; iii) many supplements are available as mixtures of different substances, confounding the effects of individual components; iv) in different products, the same substance is often present at different doses; and vi) patients who are likely to have a condition of oxidative stress could find benefit from the use of substances with an antioxidant effect.

However, it still incompletely known which molecule reaches the testis, at which dose, and sometimes the molecular mechanism of action (21).

The aim of this study was to evaluate the potential efficacy of each DS using an adapted version of the scoring system by the American Heart Association. In particular, the effect of any ingredients was evaluated according to semen alterations.

## Evidence Acquisition

### Systematic Review of Literature

We performed the present review following the Preferred Reporting Items for Systematic Reviews and Meta-Analysis (PRISMA) statement (22).

On the website of the Italian Ministry of Health, we found 23 supplements marketed for male infertility (23). Two investigators (GP and GG) performed a systematic electronic search on Google Scholar, Embase, MEDLINE, and Cochrane Register of Controlled Trials since 2000 until September 30, 2021. The following search strategy was used: (“name of each active ingredient” AND (“supplements” OR “nutraceuticals”) AND (“fertility” OR “male reproduction” OR “semen parameters”). The references of retrieved articles together with the proceedings of relevant conferences were hand-searched in order to identify other potentially eligible studies for inclusion in the analysis missed by the initial search or any unpublished data. The literature search, assessment of inclusion and exclusion criteria, quality of studies, and extraction of data were independently undertaken and verified by two investigators (AG and FF-P). The results were then compared, and in case of discrepancies, a consensus was reached with the involvement of a third senior investigator (AV). There was no language restriction applied.

**Figure 1:**
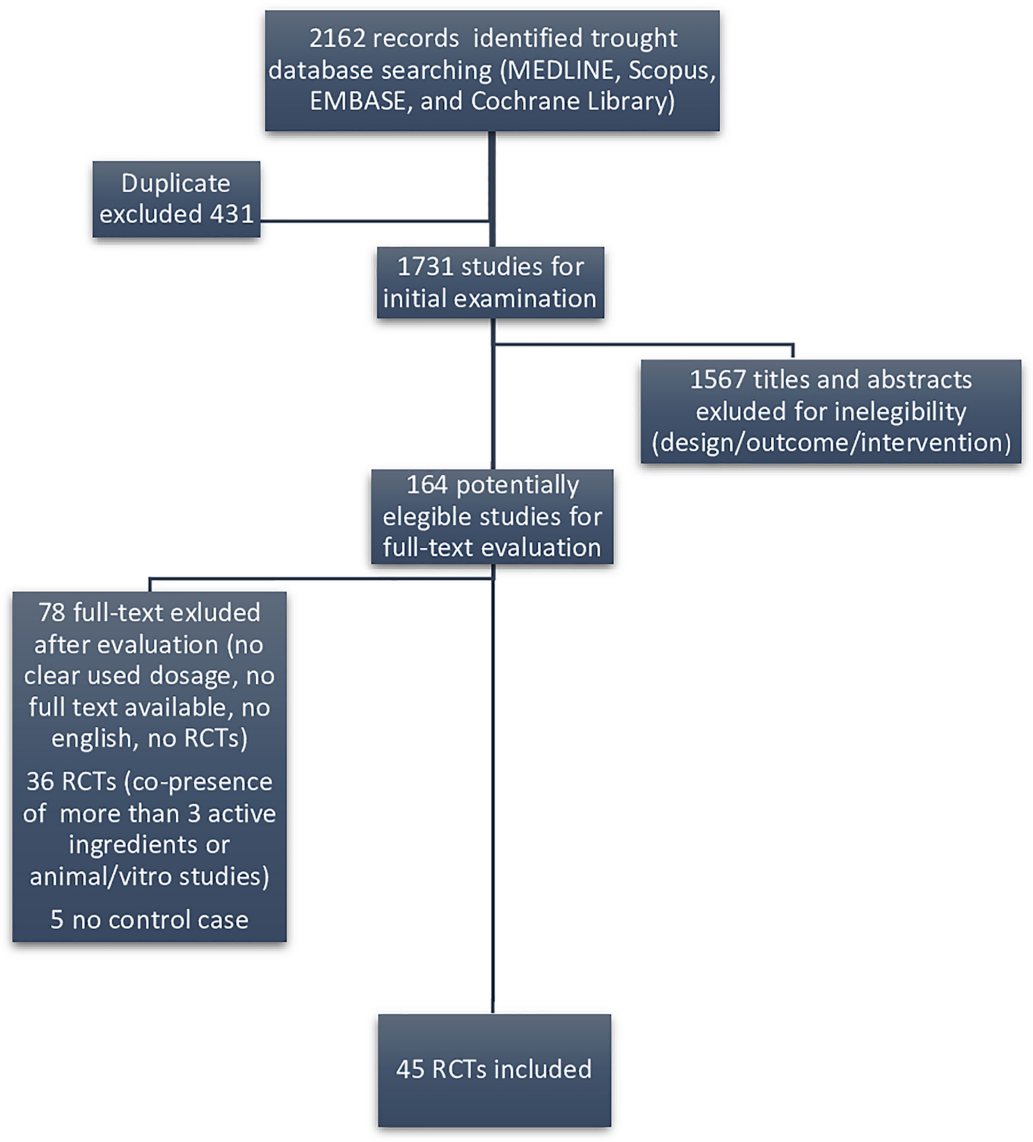
Flow diagram of eligible papers.

We considered as eligible only randomized clinical trials (RCTs) evaluating substances included in DS marketed for male infertility. **Figure 1** displays the flow diagram of the selection of eligible papers.

To ascertain the certainty of evidence, the Grading of Recommendations, Assessment, Development and Evaluations (GRADE) framework was used (24).

### Definition of Potential Active Ingredients and Minimal Effective Daily Dose

Selected articles allowed us to identify potential active ingredients (PAI) that were substances with a reported efficacy on at least one sperm parameter (sperm count, motility, morphology, DNA damage, etc.). The minimal effective daily dose (mED) was considered as the lowest effective dose reported in RCTs for each PAI, able to improve at least one sperm parameter.

### Formula to Score DS

In our previous paper (17), we suggested a formula derived from the study by Kuchakulla et al. (20) to evaluate the possible efficacy of DS based on their composition. We included both papers evaluating effective and ineffective ingredients in the improvement of sperm parameters aimed to better weigh the possible efficacy of each single molecule. The used formula was conceived as follows: ingredients contained in each supplement were classified into four categories (A, B, C, or D) based on the reported efficacy and suggested daily dose. An ingredient was assigned to category A if multiple randomized control trials showed a net positive impact, level B if just one positive RCT was found, level C if multiple RCTs showed opposing results in an indeterminate outcome, and level D if RCT showed no or even negative effect. Once a category was designated to each ingredient, a score was assigned: A = 5, B = 3, C = 1, D = −1. Subsequently, the scores were designated to each of the supplements depending on their respective composition: briefly, the score of each ingredient constituting the supplement (i.e., A = 5, B = 3, C = 1, D = −1) was summed.

Then this score was weighted for the total number of ingredients in the supplement (*N*). Finally, in order to reward those supplements with only class A and B ingredients, the relative score was multiplied by the number of class A ingredients plus half the number of class B ingredients, finally resulting in the final score of the supplement.


$$Score\;=\;\frac{\left(5A\;+3B\;+\;C-D\right)}{N}\times \left(1+A+\frac{B}{2}\right)$$

Given the distribution of the scores resulted in three main clusters, we classified DS into three categories, resembling the potential efficacy of the ingredients: high expected efficacy (corrected score ≥ 3), low expected efficacy (1 > corrected score < 3), and no expected efficacy (corrected score ≤ 1). To obtain the whole list of DS, actually marketed in Italy for male infertility, we referred to the register available on the website of the Italian Ministry of Health, updated to 01/03/2020 [23].

### Evidence Synthesis

Among the 1,731 studies for initial examination, we identified 164 eligible papers in our systematic literature search following the exclusion of duplicate publication, after screening of the title and the abstract sections of the paper. Among those, we found that only 45 RCTs reporting their efficacy on sperm parameters were retrieved as full text, in order to be included in the systematic review (**Figure 1**).

### DS Evaluation Based on Literature and Results

The analysis of literature allowed us to evaluate the potential efficacy of ingredients. We found 18 ingredients active on at least one sperm parameter and other 18 substances without any evidence. The list of PAI, references, effect on evaluated sperm parameters, and employed daily doses are summarized in **Table 1**. In the last column, the mED with demonstrated efficacy of each substance is reported. In some studies, marked with an asterisk, the employed daily dose of ingredients (zinc and α-tocopherol) exceeded the UL.

**Table 1:** Active ingredients with evidence of potential efficacy, class of expected efficacy, references, efficacy on sperm parameters, employed daily dose, and minimal effective dose (mED).

Active Ingredients	References	Evaluated Sperm Parameters	Employed Daily Dose	Number Included Patients	Minimal Effective Dose (mED)
Zinc—C/D	(25)*	↑ Motility	66 mg	211	66 mg
	(26)*	↑ Concentration	66 mg	87	
	(27)*	↑ Morphology	66 mg	160	
	(28)*	↑ Motility/DNA integrity	400 mg	47	
	(29)	↓ DNA integrity	30 mg	2,370	
	(30)	↔ No effect	220 mg	83	
Selenium—B/C	(31)	↑ Motility	100 µg	69	100 µg
	(32)	↑ Concentration/motility	200 µg	468	
	(33)	↔ No effect	300 µg	42	
**Vitamin B_12_—A/B**	(34)	↑ Count	25 µg	23	25 µg
	(35)	↑ Count	1,500 µg	375	
	(36)	↑ Count	6,000 µg	39	
Folic acid—C/D	(37)	↑ Count/motility	400 µg	194	400 µg
	(25)	↑ Volume/motility	500 µg	211	
	(26)	↑ Count	500 µg	87	
	(29)	↓ DNA integrity	500 µg	2,370	
	(30)	↔ No effect	500 µg	83	
**L-arginine—A/B**	(38)	↑ Progressive motility	1.4 g	50	1.4 g
	(39)	↑ Concentration/motility	1.4 g	50	
**L-citrulline—B/C**	(40)	↑ Volume/concentration ↑ Motility/vitality	1.2 g	50	1.2 g
**α-Lipoic acid—B/C**	(41)	↑ Count/motility	600 mg	44	600 mg
L-carnitine—B/C (LC/LAC)	(42)	↑ Motility	1 g LC	212	1 g LC
	(43)	↑ Count/motility	2 g LC	30	
	(44)	↑ Concentration/motility	2 g LC	100	
	(45)	↑ Motility	3 g LC	60	
	(46)	↔ No effect	2 g LC + 1 g LAC	21	
**N-acetyl cysteine—A/B (NAC)**	(47)	↑ Motility/DNA integrity	600 mg	120	600 mg
	(32)	↑ Motility/morphology	600 mg	468	
Coenzyme Q10—C/D	(48)	↑ Motility	200 mg	47	200 mg
	(49)	↑ Count/motility	200 mg	228	
	(50)	↑ Concentration/morphology	300 mg	212	
	(48)	↔ No effect	200 mg	47	
**Astaxanthin—B/C**	(51)	↑ Motility	16 mg	30	16 mg
**D-aspartic acid—B/C (DAA)**	(52)	↑ Count/motility	2.6 g	60	2.6 g
*Tribulus terrestris* DE—B/C	(53)	↑ Count/motility	750 mg	66	750 mg
	(54)	↑ Morphology/motility	1,500 mg	30	
	(55)	↑ Count/motility	6,000 mg	63	
	(56)	↔ No effect	750 mg	30	
**Inositol—A/B**	(37)	↑ Count/motility	4 g	194	4 g
	(57)	↑ Count	4 g	62	
α-Tocopherol—C/D	(28)	↑ Motility/morphology	20 mg	47	20 mg
	(58)	↑ Motility/lipid stability	300 mg	87	
	(59)	↑ DNA integrity	1,000 mg	64	
	(60)	↔ No effect	300 mg	50	
	(61)	↔ No effect	600 mg	30	
	(62)	↔ No effect	800 mg	31	
Vitamin C—B/C	(63)	↑ Concentration/motility	0.5 g	115	0.5 g
	(59)	↑ DNA integrity	1 g	64	
	(62)	↔ No effect	1 g	31	
EPA + DHA—B/C	(64)	↑ Concentration/motility	0.72 g + 0.48 g	238	DHA 0.48 g
	(65)	↑ DNA integrity	0.12 g + 1 g	46	
	(66)	↑ DNA integrity	1.5 g DHA	74	
	(67)	↔ No effect	400/800 mg DHA	28	
**Lycopene—B/C**	(68)	↑ Count/motility	25 mg	44	25 mg

The letters (A/B/C/D) after each active ingredient refer to the categories of the reported efficacy. In the various supplements, they can get the highest or lowest category, respectively, based on whether they are present reaching the mED or not.

LC, L-carnitine; LAC, acetyl L-carnitine; EPA, eicosapentaenoic acid; DE, dry extract; DHA, docosahexaenoic acid; ↑, improved; ↔, no effect; ↓, employed. Ingredients in bold have only studies showing positive effect.

*Employed daily dose of ingredients exceeded the UL.

Excluding astaxanthin, D-aspartic acid, and lycopene, each having just one reference, the evidence of efficacy of the other ingredients was supported by at least two RCTs. Half of the PAI had at least one study showing no or even negative effects on sperm parameters. Regarding zinc, folic acid, CQ10, and α-tocopherol, we found more than one study showing no or even negative effects.

In particular, for α-tocopherol, we found six studies: three showed a positive effect and three showed no effect. Nine ingredients (evidenced in bold in **Table 1**) were evaluated only in studies showing a positive effect. However, five substances of this group had just one paper supporting their efficacy.

The characteristics of the 23 DS are summarized in **Table 2**. In this table, compositions, recommended daily dose for each ingredient, grade of evidence, and score of efficacy are reported for each DS. A total of 36 ingredients were used by manufacturers in the DS. All the evaluated supplements were mixtures ranging from 2 up to 17 substances. Fifteen out of 23 supplements (65.2%), marked with an asterisk, contained at least 1 ingredient without evidence of efficacy on sperm parameters (evidence by cursive in **Table 2**). In 21 formulations, there were ingredients dosed below mED. In particular, one supplement (DS 12) counted 17 ingredients, of which 7 were without proven efficacy and 13 dosed below mED. Two DS (2 and 21) contained only ingredients with demonstrated efficacy and satisfying mED. DS 9 had the zinc dosed at the UL (40 mg/day). Among the DS, the most used ingredient was zinc, followed by selenium, arginine, coenzyme Q, folic acid, and carnitine. These six molecules were used in more than 60% of formulations.

**Table 2:** List of dietary supplements (DS), their composition, and score of expected efficacy.

Active Ingredients	DS 1		DS 2		DS 3*		DS 4*		DS 5*		DS 6*		DS 7	
	S = 0.8		*S* = 1.5		*S* = 1.9		*S* = 0.2		*S* = 2.8		*S* = 0.8		*S* = 2.7	
	Daily Dose	EV	Daily Dose	EV	Daily Dose	EV	Daily Dose	EV	Daily Dose	EV	Daily Dose	EV	Daily Dose	EV
Zinc	7.5 mg	D	10 mg	D	12.5 mg	D	1.5 mg	D			13 mg	D		
Selenium	60 µg	C			83 µg	C					30 µg	C	55 µg	C
Vitamin B_12_							33 mg	A						
Folic acid	200 µg	D					400 µg	C			400 µg	C	200 µg	D
L-arginine	100 mg	B			1,000 mg	B			2,500 mg	A	125 mg	B	30 mg	B
L-citrulline														
α-Lipoic acid							50 mg	C						
L-carnitine					1,000 mg	B					200 mg	C	30 mg	C
N-acetyl cysteine (NAC)														
Coenzyme Q10	10 mg	D	200 mg	C	10 mg	D			200 mg	C	7.5 mg	D		
Astaxanthin	15 mg	C												
D-aspartic acid (DAA)			2,660 mg	B										
*Tribulus terrestris* DE					800 mg	B								
Inositol													1,000 mg	B
α-Tocopherol	30 mg	C			12 mg	D			30 mg	C	36 mg	C	30 mg	C
Vitamin C	60 mg	C							180 mg	C				
DHA														
Lycopene					15 mg	C								
*Astragalus DE*					300 mg	D								
*Damiana DE*														
*Nettle DE*														
*Catuba DE*														
*Ecklonia bicyclis DE*														
*L-taurine*									500 mg	D				
*Glutathione*							30 mg	D			40 mg	D		
*Glucosamine*														
*SOD*							154 UI	D						
*Vitamin D_3_*														
*Vitamin B_1_*														
*Vitamin B_2_*							25 mg	D						
*Vitamin B_3_*							36 mg	D						
*Vitamin B_5_*														
*Vitamin B_6_*							9.5 mg	D						
*Biotin*														
*Manganese*														
*Resveratrol*														
Active Ingredients	DS 8*		DS 9*		DS 10		DS 11*		DS 12*		DS 13		DS 14*	
	*S* = 0.90		*S* = 0.80		*S* = 1.50		*S* = 0.75		*S* = 0.70		*S* = 5.60		*S* = 2.50	
	Daily Dose	EV	Daily Dose	EV	Daily Dose	EV	Daily Dose	EV	Daily Dose	EV	Daily Dose	EV	Daily Dose	EV
Zinc			40 mg	D	12.5 mg	D			10 mg	D			15 mg	D
Selenium			60 µg	C					55 µg	C	55 µg	C	83 µg	C
Vitamin B_12_									2.5 µg	B			5 µg	B
Folic acid			800 µg	C					400 µg	C	200 µg	D		
L-arginine	200 mg	B	250 mg	B	100 mg	B					30 mg	B		
L-citrulline					800 mg	C								
α-Lipoic acid									300 mg	C			800 mg	B
L-carnitine	200 mg	C	400 mg	C					500 mg	C	30 mg	C		
N-acetyl cysteine (NAC)									300 mg	B	600 mg	A		
Coenzyme Q10	15 mg	D	15 mg	D	100 mg	D			20 mg	D			200 mg	C
Astaxanthin														
D-aspartic acid (DAA)					80 mg	C								
*Tribulus terrestris* DE							300 mg	C						
Inositol							1,000 mg	B	100 mg	B	1,000 mg	B	1,000 mg	B
α-Tocopherol	30 mg	C	120 mg	C	30 mg	C					30 mg	C		
Vitamin C														
DHA														
Lycopene									4 mg	C				
*Astragalus DE*														
*Damiana DE*														
*Nettle DE*														
*Catuba DE*	50 mg	D												
*Ecklonia bicyclis DE*							200 mg	D						
*L-taurine*														
*Glutathione*			80 mg	D										
*Glucosamine*							150 mg	D						
*SOD*														
*Vitamin D_3_*														
*Vitamin B_1_*									1.1 mg	D				
*Vitamin B_2_*									1.4 mg	D			2.8 mg	D
*Vitamin B_3_*									16 mg	D				
*Vitamin B_5_*									6 mg	D				
*Vitamin B_6_*									1.4 mg	D			2.8 mg	D
*Biotin*									100 µg	D				
*Manganese*									2 mg	D				
*Resveratrol*														
Active ingredients	DS 15*		DS 16		DS 17*		DS 18*		DS 19		DS 20*		DS 21	
	*S* = 2.10		*S* = 2.00		*S* = 3.70		*S* = 0.70		*S* = 0.00		*S* = 2.00		*S* = 6.00	
	Daily Dose	EV	Daily Dose	EV	Daily Dose	EV	Daily Dose	EV	Daily Dose	EV	Daily Dose	EV	Daily Dose	EV
Zinc	15 mg	D	22.5 mg	D	10 mg	D	10 mg	D	6.5 mg	D	10 mg	D		
Selenium	50 µg	C			80 µg	C	50 µg	C			55 µg	C		
Vitamin B_12_	2.5 µg	B					1.5 µg	B						
Folic acid	400 µg	C	300 µg	D	200 µg	D	200 µg	D					400 µg	C
L-arginine			2,500 mg	A			200 mg	B			30 mg	B		
L-citrulline	3,000 mg	B							200 mg	C				
α-Lipoic acid														
L-carnitine	1,000 mg	B	200 mg	C			400 mg	C			44.7 mg	C		
N-acetyl cysteine (NAC)					600 mg	A								
Coenzyme Q10	200 mg	C					100 mg	D	90 mg	D				
Astaxanthin					16 mg	B	10 mg	C						
D-aspartic acid (DAA)									1,000 mg	C				
*Tribulus terrestris* DE														
Inositol					50 mg	B					500 mg	B	4,000 mg	A
α-Tocopherol	40 mg	C	30 mg	C			12 mg	D						
Vitamin C	80 mg	C					100 mg	C			180 mg	C		
DHA					100 mg	C								
Lycopene	10 mg	C												
*Astragalus DE*														
*Damiana DE*	400 mg	D												
*Nettle DE*	300 mg	D												
*Catuba DE*														
*Ecklonia bicyclis DE*														
*L-taurine*											300 mg	D		
*Glutathione*	40 mg	D					40 mg	D						
*Glucosamine*														
*SOD*					150 mg	D								
*Vitamin D_3_*							3.75 µg	D						
*Vitamin B_1_*														
*Vitamin B_2_*														
*Vitamin B_3_*														
*Vitamin B_5_*														
*Vitamin B_6_*														
*Biotin*														
*Manganese*														
*Resveratrol*														
Active Ingredients	DS 22*		DS 23*		DS 24	
	*S* = 0.90		*S* = 0.30		*S* = 0.70	
	Daily Dose	EV	Daily Dose	EV	Daily Dose	EV
Zinc	15 mg	D			15 mg	D
Selenium	100 µg	C			55 µg	C
Vitamin B_12_	5 µg	B	2.5 µg	B	7.5 µg	B
Folic acid	400 µg	C	400 µg	C	600 µg	C
L-arginine					415 mg	B
L-citrulline						
α-Lipoic acid						
L-carnitine					340 mg	C
N-acetyl cysteine (NAC)						
Coenzyme Q10	100 mg	D			30 mg	D
Astaxanthin	4 mg	C				
D-aspartic acid (DAA)						
*Tribulus terrestris* DE						
Inositol						
α-Tocopherol	60 mg	C			12 mg	D
Vitamin C	80 mg	C			240 mg	C
DHA	235 mg	C				
Lycopene						
*Astragalus DE*						
*Damiana DE*						
*Nettle DE*						
*Catuba DE*						
*Ecklonia bicyclis DE*						
*L-taurine*						
*Glutathione*	30 mg	D				
*Glucosamine*						
*SOD*						
*Vitamin D_3_*			25 µg	D	5 µg	D
*Vitamin B_1_*						
*Vitamin B_2_*					1.4 mg	D
*Vitamin B_3_*						
*Vitamin B_5_*						
*Vitamin B_6_*			1.4 mg	D	4.2 mg	D
*Biotin*						
*Manganese*						
*Resveratrol*			150 mg	D		

Ingredients without proven efficacy are in cursive.

S, score of the expected efficacy of the supplement; EV, efficacy value of active ingredients in relation to literature and achievement of mED; SOD, superoxide dismutase.

*DS containing at list one ingredient without evidence of efficacy.

**Figure 2:**
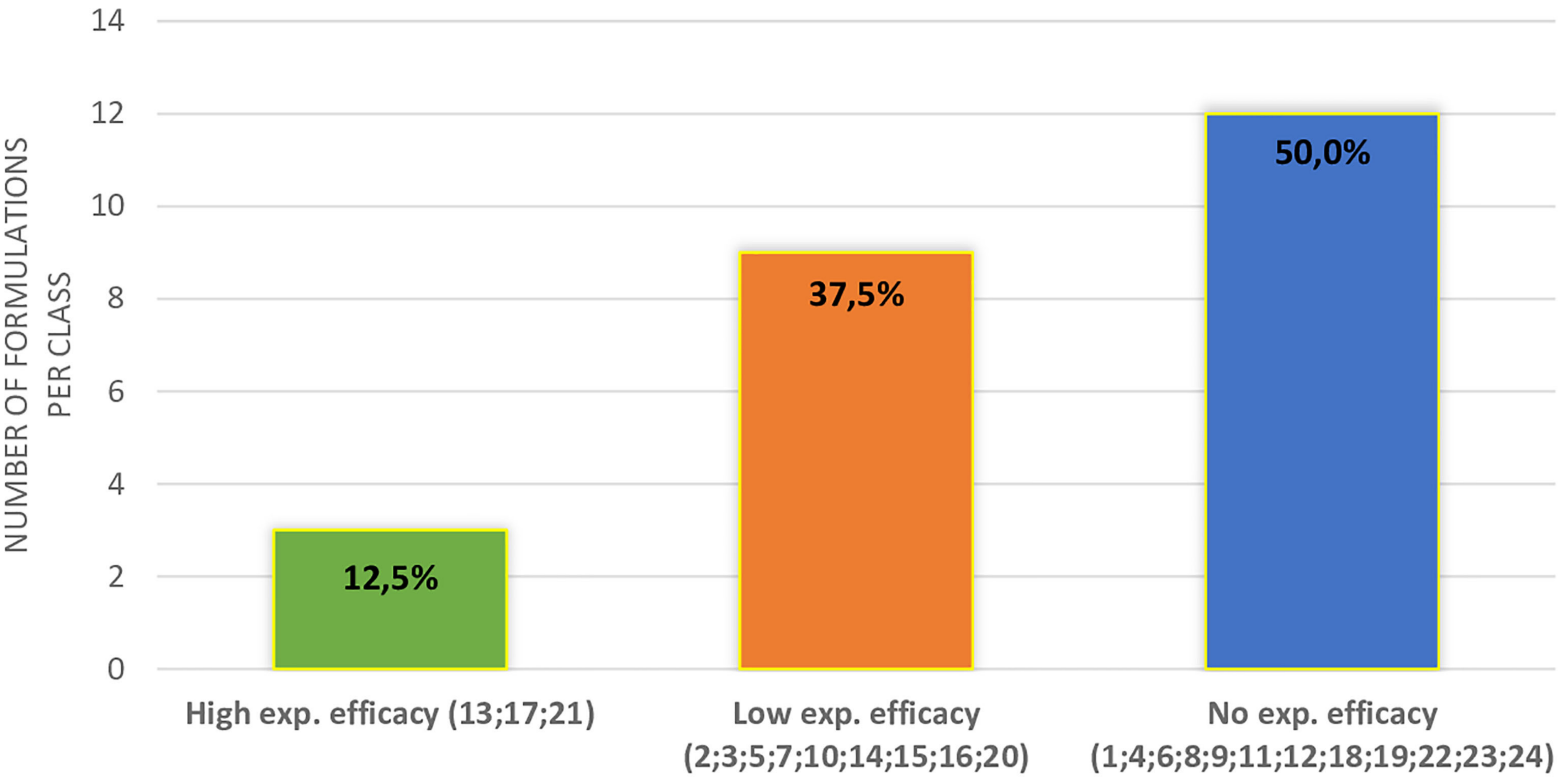
Distribution of different supplements (indicated by the number) in classes of expected efficacy.

In **Figure 2**, the distribution of supplements divided into three classes of expected efficacy based on their corrected scores is reported. Three out of 23 supplements (12.5%) resulted in the higher efficacy group and 9 (37.5%) in the lower efficacy group. The remaining 12 DS (50.0%) were expected to have no efficacy. In **Table 3**, active ingredients and DS were grouped based on the supposed efficacy on specific seminal targets (count, total motility, morphology, or DNA integrity). The most part of the ingredients showed a positive effect on sperm count and total motility. Interestingly, zinc showed evidence of efficacy on all the sperm parameters here considered. In the lower part of the table, DS were grouped in relation to their expected efficacy on specific sperm parameters, matching the corrected score and contained ingredients.

**Table 3:** Effect of active ingredients on specific sperm parameters (in the lower part of the table, DS with higher expected efficacy on each sperm parameter are listed).

	Effect on Sperm Parameters			
	Count	Total Motility	Morphology	DNA Damage
Active ingredients	Zinc Selenium Folic acid Vitamin B_12_ Folic acid L-citrulline L-arginine α-Lipoic acid L-carnitine C-Q10 DAA TT Inositol Vitamin C DHA Lycopene	Zinc Selenium Folic acid L-arginine L-citrulline α-Lipoic acid L-carnitine NAC C-Q10 Astaxanthin DAA TT Inositol α-Tocopherol Vitamin C Lycopene DHA	Zinc NAC Coenzyme Q10 TT α-Tocopherol	Zinc NAC α-Tocopherol Vitamin C DHA
Number of DS with evidence of efficacy on the parameters	21; 14; 15	17; 14; 5	13; 17; 5	13; 17; 15

## Discussion

The use of supplements in the enhancement of male fertility is as interesting and promising topic as it is still much debated. However, most trials used unsatisfactory methodology due to lack of a control group, lack of randomized and placebo-controlled well-designed study, use of many different doses of ingredients, evaluation of different outcomes, small number of included patients, etc. In fact, in the trials concerning male infertility, there is too much focus on the seminological characteristics without taking into consideration the female outcomes and also the pregnancy rate, which should always be our target (69, 70). Moreover, many clinical studies were sponsored and analyzed mixtures of many ingredients without evidence of clinical efficacy. However, these supplements are frequently prescribed in clinical practice to infertile patients, and sometimes, subjects seeking fertility spontaneously purchase these products since medical prescription is unnecessary (70). In 2019, the Italian market of supplements generated 3.7 billion euros, with an increase of +4.3% compared to 2018 (71).

In this study, we developed a new approach to evaluate DS marketed for male infertility. By a literature review, aimed to understand the clinical efficacy of each ingredient in the improvement of sperm parameters, we developed a new formula able to weigh the potential efficacy of DS. In a previous study (70), we used a similar approach to evaluate the DS for female infertility. Using the same formula, here, we included only RCTs both showing positive, none, and even negative effects of each ingredient. Moreover, we related the potential efficacy of substances on each sperm parameter. Using this method, we observed that the composition of DS marketed in Italy for male infertility is little supported by scientific evidence. In fact, most products evaluated in this study contained a huge number of ingredients, up to 17 different substances in the same supplement, frequently below the mED. A relevant issue consists in the presence of many ingredients at low dosages and/or without any evidence of efficacy. Some substances without evidence of efficacy in the literature, such as vitamin D_3_, taurine, B group vitamins, glucosamine, glutathione, various enzymes, and non-standardized dry herbal extracts, were widely present in DS for male infertility (69, 70). This evidence reflects the lack of knowledge in this field of reproductive medicine by manufacturers.

Several studies demonstrated that some substances such as omega 3 fatty acid, lycopene, and *Tribulus* have a positive effect on sperm parameters (sperm motility, number, morphology, DNA integrity, and mitochondrial function) (72–74). However, the mechanism of actions was documented for selenium (75), zinc (76, 77), and carnitine (78), but just supposed for other ingredients or still unknown in most cases. The synergistic effect of more ingredients can also be supposed. Therefore, the benefit that is obtained following the use of DS, which are always combinations of substances, is likely to be given by an overall synergistic effect. For example, the combinations of vitamin B_12_ and folic acid could have a positive effect on DNA integrity, able to improve homocysteine metabolism (79, 80).

Some ingredients, in particular amino acids, or their derivatives such as arginine and carnitine, were present in most DS aimed to improve sperm parameters and ameliorate spermatogenic process. However, these substances need to be administered at doses close to grams to be effective (81, 82). In contrast, our data demonstrate that ingredients are used even in doses 10 times below mED. In the case of arginine, the dosage should take into account the incomplete intestinal absorption and the liver metabolism which strongly reduce its bioavailability in the reproductive system (83, 84).

Using the new formula, proposed here, we scored, for the first time, the possible efficacy of each DS based on the number of ingredients, their reported or no reported efficacy, their effective or no effective dose, and their level of action at the seminal level. Only a few DS (3 products) resulted in the highest scoring level, while most products (21 DS) fell in the intermediate or lower level. This observation should be taken into account by manufacturers in order to align more and more the DS market according to scientific evidence to conceive more effective DS formulations. Despite actual literature could help to conceive better DS, it is largely insufficient to better drive clinicians on the tailored choice of the DS regarding the specific alteration of an infertile patient. This gap derives from the lack of accuracy in the diagnosis of infertility cause, observed in most clinical studies. In fact, their design often does not consider the cause that underlies sperm abnormalities. It is well known that the same sperm alteration can be induced by different causes (85). For example, a reduction of sperm motility is related both to testicular impairment, semen infections, or inflammation of accessory glands or varicocele (86). The same example can be performed for sperm count, morphology, DNA integrity, and other sperm parameters (87). For this reason, RCTs should be performed in populations of infertile patients stratified according to the etiology of infertility and not only based on seminal parameters. Moreover, to reach an effective dietary supplementation, aimed to improve sperm parameters, the mechanism of action and the effective dose of each ingredient should be clearly elucidated (88–90). With this aim, many and well-designed *in-vitro* and *in-vivo* studies are needed.

The choice to include in this review only evidence from RCTs was made in order to minimize bias in the critical evaluation of DS, in order to have for each active ingredient analyzed studies that have reported a positive effect but also studies that have shown a negative or no effect.

The main limitation of this study in the restricted focus of research on DS based on the Italian market, and the most analyzed RCTs are based on combinations of more than one substance, so it cannot be excluded that what is obtained is the result of an interaction (both positive and negative) between the different substances used together.

### Conclusions

In the light of our findings, we raise three final considerations: i) the Italian market of DS for male infertility offers products with potential efficacy in the improvement of sperm parameters but also many with uncertain effects; ii) the actual literature is poor of well-designed studies on PAI investigating their mechanisms of action and effective dose in different pathological conditions; and iii) based on current literature, our study can help in the choice of DS and PAI that are more likely to be effective on specific sperm alterations.

Our critical analysis suggests a rational strategy for a tailored use of DS in male infertility.

## Data Availability Statement

The original contributions presented in the study are included in the article/supplementary material. Further inquiries can be directed to the corresponding author.

## Author Contributions

AG, GP, and FF-P contributed to the concept/design of the research and acquisition/analysis of the literature data. AG, GP, and FF-P equally contributed to and drafted the manuscript. AN contributed to the concept and performed the data analyses. LT, AV, GG, and CF critically revised the paper for important intellectual content. All authors revised and approved the final manuscript and agreed to be fully accountable for ensuring the integrity and accuracy of the work. All authors had full access to all the data in the study and took responsibility for the integrity of the study and the accuracy of the analysis.

## Conflict of Interest

The authors declare that the research was conducted in the absence of any commercial or financial relationships that could be construed as a potential conflict of interest.

## Publisher’s Note

All claims expressed in this article are solely those of the authors and do not necessarily represent those of their affiliated organizations, or those of the publisher, the editors and the reviewers. Any product that may be evaluated in this article, or claim that may be made by its manufacturer, is not guaranteed or endorsed by the publisher.
